# Machine learning-assisted detection of canine mammary tumors using serum autoantibody signatures

**DOI:** 10.1080/01652176.2026.2617470

**Published:** 2026-01-21

**Authors:** Bluest Lan, Chia-Yu Chang, Shin-Wu Liu, Chih-Ching Wu, Kuan-Ming Lai, Hao-Ping Liu

**Affiliations:** a Department of Mechanical Engineering, College of Engineering, National Chung Hsing University, Taichung, Taiwan; b Department of Veterinary Medicine, College of Veterinary Medicine, National Chung Hsing University, Taichung, Taiwan; c Graduate Institute of Biomedical Sciences, College of Medicine, Chang Gung University, Taoyuan, Taiwan; d Department of Medical Biotechnology and Laboratory Science, College of Medicine, Chang Gung University, Taoyuan, Taiwan; e Department of Otolaryngology-Head & Neck Surgery, Linkou Chang Gung Memorial Hospital, Taoyuan, Taiwan; f Molecular Medicine Research Center, Chang Gung University, Taoyuan, Taiwan; g Hemato-Oncology Division Department of Internal Medicine, Changhua Christian Hospital, Changhua, Taiwan; h Biotechnology Center, National Chung Hsing University, Taichung, Taiwan

**Keywords:** Machine learning, canine mammary tumor, multiplex, serum, autoantibody, biomarker

## Abstract

Canine mammary tumors (CMTs) are the most common neoplasms in intact female dogs, yet early detection remains challenging due to the lack of clinically validated, noninvasive biomarkers. This study aimed to develop a noninvasive diagnostic model for CMT detection by integrating serum autoantibody biomarkers with machine learning. Serum samples from 154 dogs with mammary tumors (31 benign, 123 malignant) and 39 healthy controls were analyzed using a custom multiplex immunoassay detecting autoantibodies against AGR2, HAPLN1, IGFBP5, and TYMS, normalized to anti-BSA levels. Median fluorescence intensity (MFI), standardized autoantibody ratios, and their combination, together with clinical variables, were used to train random forest classifiers. The model based on standardized autoantibody ratios achieved the best performance, with an AUC of 0.79 (sensitivity 75.3%, specificity 74.4%) for overall CMT detection; 0.78 (92.7%, 61.5%) for malignant CMTs; and 0.77 (82.2%, 71.8%) for early-stagemalignancies. Assuming a CMT prevalence of 0.5 in the hospital-referred population, the positive and negative predictive values ranged from 0.74–0.75 and 0.75–0.91, respectively. This proof-of-concept study demonstrates that a machine learning-assisted multiplex autoantibody assay offers a feasible noninvasive approach for CMT detection. Further validation in larger, independent cohorts is warranted to support clinical translation in veterinary oncology.

## Introduction

Canine mammary tumors (CMTs) are among the most prevalent neoplasms in intact female dogs, accounting for up to 50% of all diagnosed tumors in this population (Gray et al. 2020; Aupperle-Lellbach et al. 2022). Approximately half of these cases are malignant, with a subset exhibiting aggressive clinical behavior and poor prognosis. Current clinical management relies primarily on surgical excision, with histopathological evaluation serving as the diagnostic gold standard. However, this approach is inherently invasive and often delayed until the disease has progressed to advanced stages, thereby limiting opportunities for early intervention and optimal therapeutic outcomes.

To address this limitation, recent studies have explored serum-based autoantibody biomarkers as minimally invasive alternatives for cancer detection (Chang et al. 2021; Zhang et al. 2022). Tumor-associated autoantibodies, which reflect the host immune responses to aberrant tumor antigens, can be detected with high sensitivity and reproducibility using multiplex immunoassays, facilitating large-scale screening (Hussain et al. 2018; Wu et al. 2025). In our previous work, we developed a fluorescence bead-based suspension array to quantify four CMT-associated serum autoantibodies targeting anterior gradient 2 (AGR2), hyaluronan and proteoglycan link protein 1 (HAPLN1), insulin-like growth factor-binding protein 5 (IGFBP5), and thymidylate synthetase (TYMS)—proteins overexpressed in CMT tissues (Wu et al. 2019) and capable of eliciting measurable humoral responses (Chang et al. 2021; Yuan et al. 2022). This four-autoantibody panel achieved a sensitivity of 50.4% and a specificity of 90% for detecting malignant CMT (Wu et al. 2025).

Despite these findings, interpreting multiplex biomarker profiles remains analytically challenging due to high dimensionality and biological variability. Machine learning algorithms, particularly ensemble methods such as random forests (RFs), have demonstrated robust performance in biomedical classification tasks owing to their ability to integrate complex datasets and identify subtle, non-linear patterns (Felici et al. 2025). While deep neural networks offer powerful modeling capabilities, they often require large training datasets, are computationally intensive, and operate as “black boxes”, limiting interpretability (Gaur et al. 2021)—a key consideration in biomedical applications where model transparency is critical for biological and clinical insight. In contrast, RFs provide a balance between predictive accuracy and interpretability, yielding feature importance metrics that aid biological inference while maintaining robustness across heterogenous datasets (Lan and Lai 2025). When combined with rigorous cross-validation, RF algorithms can produce accurate and generalizable predictions, thereby enhancing the diagnostic utility of multiple biomarker panels (Banaei et al. 2019; Nené et al. 2023; Xavier et al. 2024; Yin et al. 2024; Felici et al. 2025).

In this study, we aimed to develop a machine learning-based model for CMT detection by integrating serum levels of four autoantibodies (anti-AGR2, anti-HAPLN1, anti-IGFBP5, anti-TYMS) with clinical variables including age, body weight, spay status, and breed. Using RF classifiers with k-fold cross-validation, we evaluated diagnostic performance of this combined biomarker-clinical model and demonstrated the feasibility of this noninvasive approach. Our results underscore the potential of machine learning to enhance biomarker interpretability and support early-stage CMT detection in veterinary oncology.

## Materials and methods

### Sample collection and classification

Serum samples were collected from client-owned female dogs presented to the Veterinary Medical Teaching Hospital (VMTH) at National Chung Hsing University (NCHU), Taichung, Taiwan, between 2018 and 2022. The study cohort included dogs with histologically confirmed mammary tumors and clinically healthy, age-matched controls without any history or evidence of neoplastic or systemic disease. Healthy dogs were recruited *via* a free health examination program and verified to be clinically healthy based on comprehensive physical examinations and serological evaluations. Tumor cases were classified as benign or malignant according to established histopathological criteria (Goldschmidt et al. 2011; Sorenmo et al. 2011; Tavasoly et al. 2013), as previously described (Wu et al. 2025). Eligibility for inclusion in the tumor group required that dogs had received no prior chemotherapy, surgical intervention or other systemic treatments before sample collection.

A total of 193 serum samples were analyzed, comprising 39 healthy controls, 31 dogs with benign mammary tumors, and 123 with malignant mammary tumors (59 at Stage I, 19 at Stage II, 22 at Stage III, 16 at Stage IV, and 12 at Stage V). All procedures were approved by the Institutional Animal Care and Use Committee (IACUC) of NCHU (Code: 109-002). Informed consent was obtained from all owners prior to sample collection.

### Quantification of serum autoantibody levels

Serum autoantibodies were detected using beads coated with affinity-purified antigens, including AGR2, HAPLN1, IGFBP5, and TYMS (Wu et al. 2025). Autoantibody signals were measured with the Bio-Plex 200 system (Bio-Rad Laboratories) and analyzed using Bio-Plex Manager software (version 4.2), as described previously (Wu et al. 2025). Median fluorescence intensity (MFI) values were recorded for each antigen-bead pair.

To correct for inter-sample variability in immunoreactivity, MFI values were normalized to signals from bovine serum albumin (BSA)-coated beads. Given the high conservation of albumins across mammals and previous evidence of cross-reactivity (Liu et al. 2023), anti-BSA signals were used to approximate baseline immune activity and nonspecific binding. Final results were expressed as standardized autoantibody-to-BSA ratios (e.g. anti-AGR2/anti-BSA) for each sample to enhance comparability across individuals.

### Data processing

Raw MFI data and standardized autoantibody ratios were preprocessed using custom Python scripts. Prior to model training, the dataset was restructured, and randomly shuffled to minimize potential ordering bias. Categorical variables, including age, spay status, body weight, and breed, were numerically encoded to ensure compatibility with the machine learning algorithm. Only samples containing complete biomarkers and clinical data were retained for downstream analyses.

### Machine learning pipeline

To classify samples based on serum biomarker profiles and clinical variables, a random forest (RF) classifier was constructed. RF is an ensemble learning algorithm that generates multiple decision trees using the Classification and Regression Tree (CART) method and aggregates their predictions to improve accuracy and generalizability (Krzywinski and Altman 2017). In contrast to entropy-based algorithms such as Iterative Dichotomiser 3 (ID3), CART employs the Gini Index as a computationally efficient impurity metric suitable for continuous variables and binary classification (Loh 2014).

The Gini Index measures node impurity and is defined for a given node t as:

$$G(t)=1-\sum_{i=1}^{C}p_{i}^{2},$$

where C is the number of classes, and $p_{i}$ denotes the proportion of samples in class i at node t. A lower Gini value indicates higher node purity, and the optimal split maximizes impurity reduction across child nodes.

By averaging predictions from multiple decorrelated CART trees, RF minimizes variance and mitigates overfitting, making it particularly suitable for complex and heterogeneous biomedical datasets. The RF model in this study was trained to classify: (1) all CMTs (benign + malignant) *vs*. healthy controls; (2) benign CMTs *vs*. healthy controls; (3) malignant CMTs *vs*. healthy controls; (4) early-stage (Stages I–II) malignant CMTs *vs*. healthy controls; (5) benign *vs*. malignant CMTs; (6) benign *vs*. early-stage (Stages I-II) malignant CMTs.

To ensure generalizability and prevent overfitting, k-fold cross-validation was applied (Lan et al. 2022). The dataset was randomly partitioned into k subsets (folds); in each iteration, one-fold served as the test set, and the remaining k−1 folds were used for training. This process was repeated k times so that each fold was used once for testing. Here, k = 5 (five-fold cross-validation), corresponding to an 80/20 train-test split per iteration.

Each decision tree in the RF was trained on a bootstrapped subset of the training data, with a random subset of features considered at each split (Breiman 2001). For a given input instance x∈R, where d denotes the number of features, the ensemble prediction of the RF model is:

$${\hat{f}}_{\text{RF}}\left(x\right)=\frac{1}{B}\overset{B}{\underset{b=1}{\sum }}{\hat{f}}_{b}\left(x\right),$$

where ${\hat{f}}_{b}(x)$ is the prediction from the b-th base tree, and B is the total number of trees. Averaging predictions across trees reduces variance and enhances generalization, particularly when the base trees are high-variance estimators. The ensemble variance can be expressed as:

$$\text{Var}[{\hat{f}}_{\text{RF}}(x)]=\rho \cdot \text{Var}[{\hat{f}}_{\text{tree}}(x)]+\frac{1-\rho }{B}\cdot \text{Var}[{\hat{f}}_{\text{tree}}(x)],$$

where ρ∈[0, 1] represents the average pairwise correlation between trees. Based on empirical convergence behavior, B was set to 100 in this study. Feature influence was determined by the total reduction in Gini impurity contributed by each variable, providing a quantitative measure of its relative contribution to overall model classification.

### Performance evaluation

Model performance was assessed using standard classification metrics: accuracy, precision (positive predictive value), sensitivity (recall), specificity, and the area under the receiver operating characteristic curve (AUC-ROC). These metrics represent complementary aspects of diagnostic performance and clinical relevance:

Accuracy: proportion of correctly classified samples

Precision: proportion of true positives among all predicted positives

Sensitivity (Recall): true positive rate (TPR)

Specificity: true negative rate (TNR)

AUC-ROC: a threshold-independent measure summarizing the trade-off between sensitivity and specificity.

These metrics were calculated as follows:

$$\text{Accuracy}=\frac{\text{TP}+\text{TN}}{\text{TP}+\text{TN}+\text{FP}+\text{FN}}$$

$$\text{Precision}=\frac{\text{TP}}{\text{TP}+\text{FP}}$$

$$\text{Sensitivity }(\text{TPR})=\frac{\text{TP}}{\text{TP}+\text{FN}}$$

$$\text{Specificity }(\text{TNR})=\frac{\text{TN}}{\text{TN}+\text{FP}},$$

where TP, TN, FP, and FN denote true positives, true negatives, false positives, and false negatives, respectively.

The AUC-ROC was obtained by plotting the true positive rate (TPR) against the false positive rate (1-TNR) across all classification thresholds, providing a comprehensive evaluation of model discrimination ability. The optimal decision threshold for diagnostic classification was determined using Youden’s J statistic (*J* = *TPR* + *TNR* − 1), which maximizes the combined sensitivity and specificity to ensure both statistical robustness and clinical interpretability.

### Predictive value analysis

The positive predictive value (PPV) and negative predictive value (NPV) were estimated to further assess the clinical diagnostic performance of each model. These values were calculated from the test sensitivity (*TPR*), specificity (*TNR*), and disease prevalence (*P*) according to the following equations:

$$\text{PPV}=\frac{\text{TPR}\times \text{TNR}}{\left(\text{TPR}\times P)+(1-\text{TNR})\times (1-P\right)}$$

$$\text{NPV}=\frac{\text{TNR}\times (1-P)}{\left(1-\text{TPR})\times P+\text{TNR}\times (1-P\right)}$$

PPV represents the probability that an individual with a positive test result truly has the disease, whereas NPV indicates the probability that an individual with a negative test result is truly disease-free.

### Statistical analysis

Differences in serum autoantibody levels between experimental groups were evaluated using the nonparametric Mann–Whitney U test implemented in GraphPad Prism (version 9; GraphPad Software, San Diego, CA, USA). A two-tailed *p* value < 0.05 was considered statistically significant. All machine learning-based analyses and visualizations were carried out in Python using in-house developed scripts.

## Results

### Profiling of serum autoantibodies

A five-plex immunoassay were conducted to quantify serum levels of autoantibodies against AGR2, IGFBP5, HAPLN1, TYMS, and BSA in a total of 193 female dogs (Table 1), including 39 healthy controls, 31 dogs with benign mammary tumors, and 123 dogs with malignant mammary tumors (59 at Stage I, 19 at Stage II, 22 at Stage III, 16 at Stage IV, and 12 at Stage V).

**Table 1.** Signalment and clinical staging of dogs included in the multiplex serum autoantibody analysis (Wu et al. 2025).

Characteristics	Healthy controls	Benign CMTs	Malignant CMTs
Case number	39	31	123
Median / Range of age (years)	8 / 2‒17	8.4 / 2‒16	10 / 1‒16
Median / Mean of body weight (kg)	13.0 / 13.4	5.6 / 9.7	7.0 / 10.7
Spayed (%)	36 (92.3)	14 (45.2)	58 (47.2)
Breed			
Maltese	2	6	18
Poodle	3	5	15
Dachshund	1	5	15
Golden Retriever	3	1	7
Chihuahua	3	0	6
Shih-Tzu	1	0	6
Schnauzer	2	0	5
Beagle	1	2	4
Shiba	1	4	3
Yorkshire	1	2	3
Spitz	1	1	2
Collie	1	1	2
Bichon Frise	1	0	1
Pomenarian	1	0	1
Other pedigrees	2	2	10
Mixed	15	2	26
Stages: I / II/ III/ IV / V^a^	–	–	54 / 19 / 22 / 16 / 12

^a^ Clinical staging was determined based on the modified WHO classification system for domestic animals (Sorenmo et al. 2011).

Raw MFI values for all five autoantibodies were significantly higher in healthy controls compared to CMT groups (Table 2, upper section). The AUC values for distinguishing all CMTs from healthy controls ranged from 0.583 to 0.671 (Table S1, upper section), with sensitivity/specificity ranging from 35.9%/86.4% to 79.5%/45.5% (Table S2, upper section).

**Table 2.** Serum autoantibody levels measured by a multiplex immunoassay in healthy dogs and those with CMTs.

Serum autoantibody	Healthy controls (*n* = 39)	CMTs (*n* = 154)	*p*-value^c^	Benign CMTs (*n* = 31)	*p*-value^c^	Malignant CMTs (*n* = 123)	*p*-value^c^	Stage I-II (*n* = 73)	*p*-value^c^
MFI values^a^									
Anti-AGR2	10156.8 ± 6187.5	7567.8 ± 4610.7	0.0224	7125.4 ± 5021.6	0.0211	7618.0 ± 4489.3	0.043	7720.8 ± 4155.0	0.1058
Anti-HAPLN1	10297.4 ± 9266.1	7027.1 ± 6524.6	0.1119	7297.2 ± 6866.9	0.3002	6959.0 ± 6430.3	0.1109	6821.3 ± 6179.2	0.2029
Anti-IGFBP5	4875.7 ± 2783.7	3731.5 ± 2432.5	0.0075	3595.0 ± 2689.8	0.0124	3765.9 ± 2356.2	0.0151	3639.8 ± 1922.7	0.0161
Anti-TYMS	6046.6 ± 5903.6	4315.2 ± 4961.2	0.0283	4382.2 ± 5220.2	0.1713	4298.3 ± 4889.8	0.0264	4379.1 ± 4602.5	0.0684
Anti-BSA	9403.6 ± 9706.7	4851.2 ± 6475.1	0.0013	5466.2 ± 6885.1	0.0465	4696.2 ± 6355.2	0.0012	4711.3 ± 5938.6	0.0045
Standardized ratios (normalized with anti-BSA)^b^									
Anti-AGR2	2.2 ± 1.8	3.9 ± 3.7	0.0014	2.9 ± 2.3	0.1226	4.2 ± 4.0	0.0007	4.0 ± 3.4	0.0018
Anti-HAPLN1	1.7 ± 1.2	2.7 ± 2.0	0.0001	2.3 ± 1.6	0.0227	2.8 ± 2.1	<0.0001	2.6 ± 1.8	0.0005
Anti-IGFBP5	1.4 ± 1.5	2.3 ± 2.6	0.0058	1.7 ± 1.6	0.1876	2.4 ± 2.8	0.0034	2.2 ± 2.2	0.0098
Anti-TYMS	0.9 ± 0.5	1.4 ± 1.2	0.0003	1.2 ± 0.7	0.0517	1.5 ± 1.3	0.0002	1.4 ± 1.4	0.0007

^a^ Autoantibody levels are represented as median fluorescence intensity (MFI) values. Group data are presented as mean ± standard deviation (SD).

^b^ Standardized ratios were calculated by dividing the MFI of each autoantibody by the MFI of anti-BSA in the same sample. Group data are presented as mean ± SD.

^c^ *p*-values for comparison between CMT groups and healthy controls were determined using the Mann-Whitney test. A *p*-value < 0.05 was considered statistically significant.

After normalization to anti-BSA levels, standardized ratios of anti-AGR2, anti-HAPLN1, anti-IGFBP5, and anti-TYMS were significantly elevated in CMTs, particularly in malignant and early-stage (Stages I-II) cases, compared with healthy controls (Table 2, lower section). The corresponding AUCs for distinguishing malignant CMTs and early-stage CMTs from healthy controls ranged from 0.654 to 0.707 and 0.648 to 0.699, respectively (Table S1, lower section), with sensitivity/specificity values between 71.5%/64.1% and 89.0%/46.2% (Table S2, lower section).

Although the standardized ratios improved discriminative performance relative to raw MFI values, the AUCs remained in the moderate range (0.642‒0.707), suggesting that a multivariate classification approach could better capture disease-associated patterns.

### Integration of machine learning to improve classification

**Figure 1.**
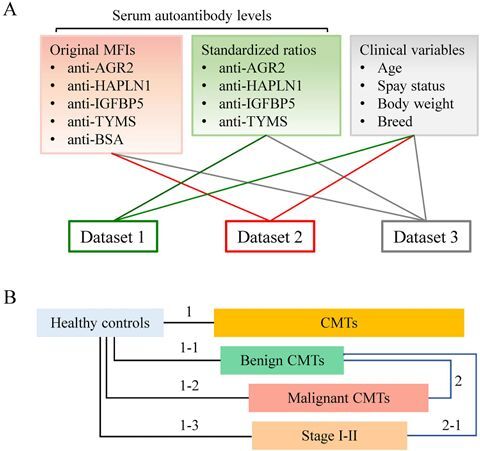
Overview of datasets and classification tasks for model training. (A) Three input datasets were constructed: Dataset 1 (standardized autoantibody ratios), Dataset 2 (original MFI values), and Dataset 3 (combined ratios and MFI values). Each dataset was integrated with clinical variables (e.g. age, body weight) for model development and evaluation. (B) Classification tasks included: (1) healthy controls (*n* = 39) *vs*. all CMTs (*n* = 154); (1–1) healthy controls *vs*. benign CMTs (*n* = 31); (1–2) healthy controls *vs*. malignant CMTs, (*n* = 123); (1–3) healthy controls *vs*. stage I–II malignant CMTs (*n* = 73); (2) benign *vs*. malignant CMTs; and (2-1) benign *vs*. stage I–II malignant CMTs.

To enhance classification accuracy, machine learning models were applied to integrate autoantibody profiles with clinical variables (Figure 1A). This strategy leveraged multivariate interactions and nonlinear relationships among features (variables) to improve the detection and stratification of benign, malignant, and early-stage malignant CMTs (Figure 1B).

**Figure 2.**
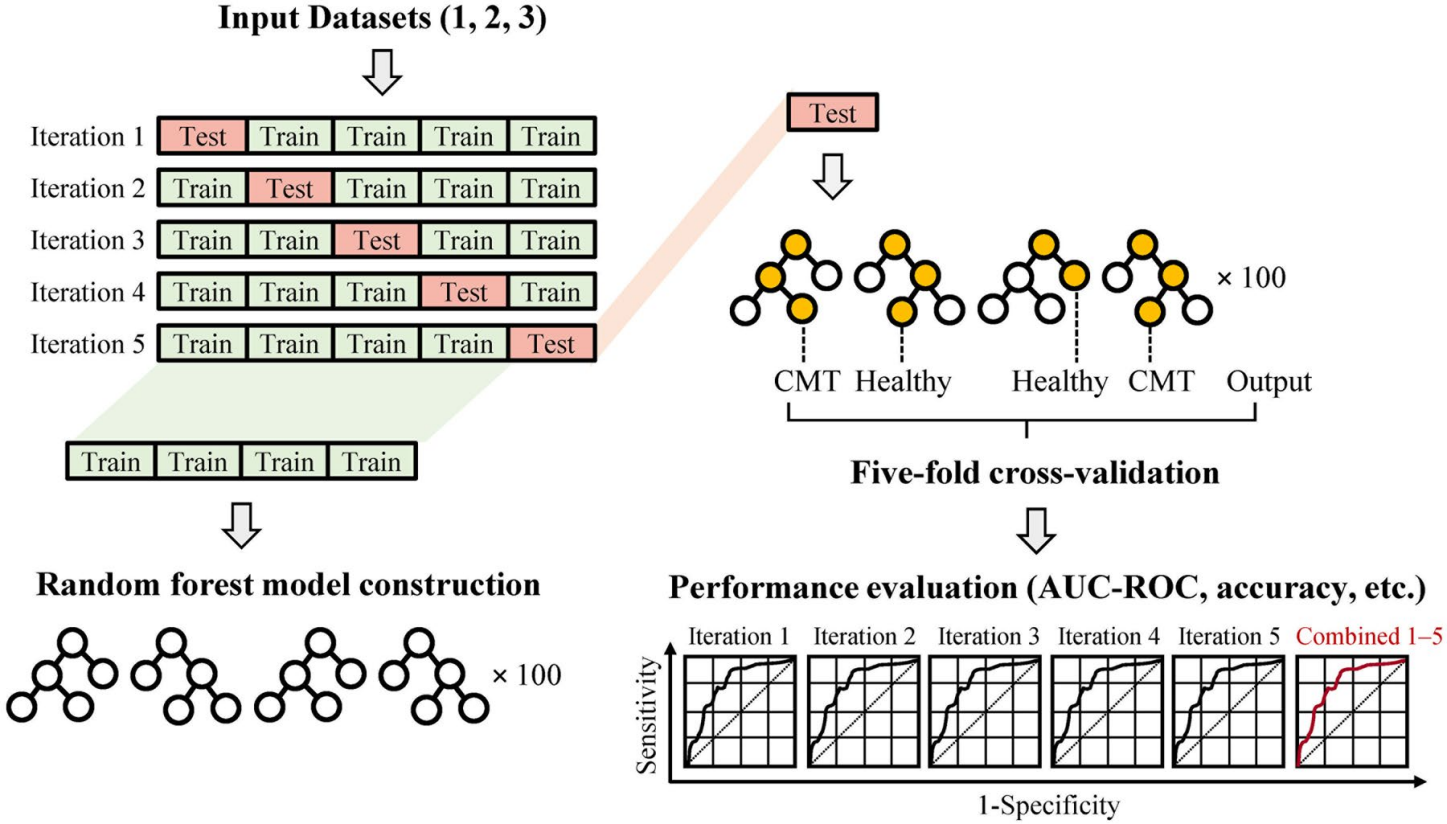
Workflow for model development and evaluation. Each dataset (Datasets 1–3) was used to train RF classifiers with a five-fold cross-validation strategy. In each fold, the data were partitioned into training and test subsets to iteratively optimize and assess model performance. Classification tasks corresponded to those described in Figure 1. Model performance was evaluated by ROC curves and AUC values derived from individual folds and combined predictions.

**Figure 3.**
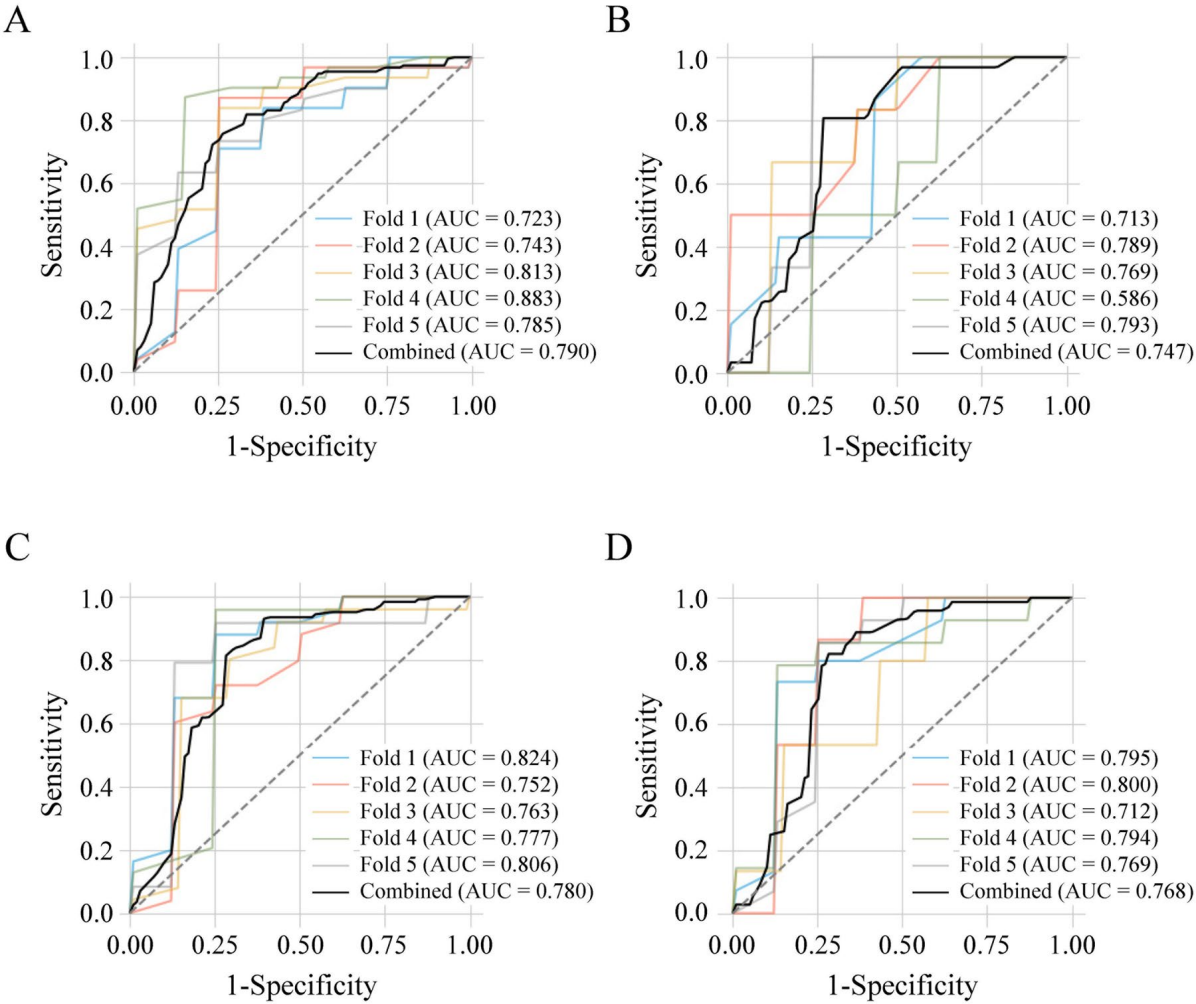
ROC curves for models trained on Dataset 1 (standardized autoantibody ratios). RF models were evaluated using five-fold cross-validation for: (A) healthy controls (*n* = 39) *vs*. all CMTs (*n* = 154) (1); (B) healthy controls *vs*. benign CMTs (*n* = 31) (1-1); (C) healthy controls *vs*. malignant CMTs (*n* = 123) (1–2); and (D) healthy controls *vs*. stage I–II malignant CMTs (*n* = 73) (1–3). Each panel displays ROC curves for individual folds (colored lines) and combined predictions (black line), with corresponding AUC values. The dashed diagonal line indicates chance-level classification.

**Figure 4.**
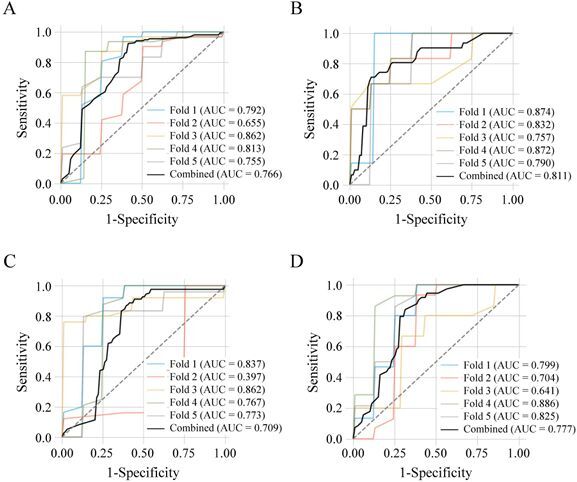
ROC curves for models trained on Dataset 2 (original MFI values). Classification tasks and cross-validation procedures were identical to those in Figure 3. Panels (A–D) correspond to: (A) healthy controls (*n* = 39) *vs*. all CMT cases (*n* = 154) (1); (B) healthy controls *vs*. benign CMTs (*n* = 31) (1-1); (C) healthy controls *vs*. malignant CMTs (*n* = 123) (1–2); and (D) healthy controls *vs*. stage I–II malignant CMTs (*n* = 73) (1–3). Colored lines represent ROC curves for individual folds, and the black line indicates the combined prediction. AUC values are provided, and the dashed diagonal line denotes random classification.

Three datasets (Datasets 1‒3) were used to train RF classifiers using a five-fold cross-validation strategy (Figure 2). For each fold, the dataset was partitioned into training and test subsets, and model performance was assessed using AUC-ROC, accuracy, and precision (Table 3, Figures 3 and 4).

**Table 3.** Evaluation of the model accuracy and performance metrics using five-fold cross-validation.

Performance metrics	Healthy controls *vs.*				Benign CMTs *vs.*	
	CMTs^a^	Benign CMTs^a^	Malignant CMTs^a^	Stages I-II CMTs^a^	Malignant CMTs^a^	Stages I-II CMTs^a^
Dataset 1 (Standardized ratios)						
AUC^b^ (range)	0.790 (0.723 ‒ 0.883)	0.747 (0.586 ‒ 0.793)	0.780 (0.752 ‒ 0.824)	0.768 (0.712 ‒ 0.8)	0.478 (0.403 ‒ 0.580)	0.524 (0.359 ‒ 0.667)
Accuracy^c^	0.751	0.757	0.852	0.786	0.727	0.596
Precision^d^	0.921	0.694	0.884	0.845	0.829	0.754
Dataset 2 (MFI values)						
AUC^b^ (range)	0.766 (0.655 ‒ 0.862)	0.811 (0.757 ‒ 0.874)	0.709 (0.397 ‒ 0.862)	0.777 (0.641 ‒ 0.886)	0.606 (0.535 ‒ 0.686)	0.602 (0.435 ‒ 0.775)
Accuracy^c^	0.855	0.800	0.802	0.786	0.461	0.587
Precision^d^	0.899	0.815	0.876	0.836	0.935	0.826
Dataset 3 (Combined standardized ratios and MFI values)						
AUC^b^ (range)	0.746 (0.668 ‒ 0.79)	0.785 (0.705 ‒ 0.926)	0.762 (0.661 ‒ 0.842)	0.744 (0.682 ‒ 0.807)	0.525 (0.454 ‒ 0.664)	0.557 (0.478 ‒ 0.661)
Accuracy^c^	0.803	0.757	0.802	0.732	0.753	0.587
Precision^d^	0.908	0.694	0.882	0.821	0.815	0.768

^a^ Clinical classifications correspond to those shown in Figure 1B: 1 (healthy vs. all CMTs), 1-1 (healthy vs. benign CMTs), 1–2 (healthy vs. malignant CMTs), 1–3 (healthy vs. stage I–II malignant CMTs), 2 (benign vs. malignant CMTs), and 2-1 (benign vs. stage I–II malignant CMTs).

^b^ Area under the curve (AUC) was calculated using combined predictions from folds 1 to 5. The reported range reflects the minimum and maximum AUC values across individual folds.

^c^ Accuracy indicates the proportion of correctly classified instances (both true positives and true negatives) among all samples.

^d^ Precision reflects the proportion of positive predictions that are true positives.

### Evaluation of the model accuracy and performance metrics

The RF model trained on Dataset 1 (standardized autoantibody ratios; Figure 3A–D) demonstrated robust performance in distinguishing CMTs from healthy controls, achieving an AUC of 0.790 (Figure 3A), accuracy of 0.751, and precision of 0.921 (Table 3, top section), with optimal sensitivity and specificity of 75.3% and 74.4%, respectively (Table 4, top section). The same model effectively detected malignant and early-stage malignant CMTs, yielding AUCs of 0.78 and 0.768 (Figure 3C–D) and sensitivity/specificity values of 92.7%/61.5% and 82.2%/71.8%, respectively (Table 4, top section). Across all malignant classification tasks, the model consistently achieved AUCs above 0.72 (Figure 3), outperforming single-marker analysis of standardized ratios (Tables S1 and S2).

**Table 4.** Diagnostic performance of the machine learning model for CMT detection.

	Healthy controls *v*s.			
Diagnostic parameters	CMTs	Benign CMTs	Malignant CMTs	Stages I-II CMTs
Dataset 1 (Standardized ratios)				
Decision threshold^a^	0.78	0.60	0.63	0.53
Sensitivity^b^	0.753	0.806	0.927	0.822
Specificity^c^	0.744	0.718	0.615	0.718
PPV^c^	0.746	0.741	0.751	0.742
NPV^c^	0.751	0.787	0.906	0.846
Dataset 2 (MFI values)				
Decision threshold^a^	0.66	0.50	0.57	0.58
Sensitivity^b^	0.922	0.710	0.862	0.836
Specificity^b^	0.590	0.872	0.615	0.692
PPV^c^	0.693	0.806	0.737	0.742
NPV^c^	0.846	0.731	0.871	0.839
Dataset 3 (Combined standardized ratios and MFI values)				
Decision threshold^a^	0.72	0.59	0.53	0.59
Sensitivity^b^	0.838	0.806	0.854	0.753
Specificity^b^	0.667	0.718	0.641	0.692
PPV^c^	0.713	0.741	0.729	0.710
NPV^c^	0.789	0.787	0.832	0.737

^a^ Decision threshold represents the predicted probability, indicating the model-estimated likelihood of a sample belonging to the positive class. The optimal threshold was determined by maximizing Youden’s J statistic to balance sensitivity and specificity.

^b^ Sensitivity and specificity were calculated using the optimal cutoff derived from Youden’s J statistic.

^c^ PPV and NPV denote the positive and negative predictive values, respectively, calculated assuming a CMT prevalence of 0.5 in the target population.

The model trained on Dataset 2 (MFI values; Figure 4A‒D) showed the highest performance for detecting benign CMTs, with an AUC of 0.811 (Figure 4B), accuracy of 0.800, and precision of 0.815 (Table 3, middle section). Corresponding sensitivity and specificity were 71.0% and 87.2%, respectively (Table 4, middle section). However, its performance was lower for distinguishing benign from malignant or early-stage malignant CMTs, with AUCs of 0.606 and 0.602, respectively (Table 3, right-middle section).

In contrast, the model trained on Dataset 3 (combined standardized ratios and MFI values) demonstrated the lowest overall performance for detecting CMTs and early-stage malignancy, with AUCs of 0.746 and 0.744 (Table 3, bottom section) and sensitivity/specificity values of 83.8%/66.7% and 75.3%/69.2%, respectively (Table 4, bottom section).

### Diagnostic performance of the machine learning models

Diagnostic performance was further assessed based on optimized decision thresholds, positive predictive value (PPV), and negative predictive value (NPV) (Table 4). Decision threshold, representing the model-estimated probability of class membership, was optimized using Youden’s J statistic to balance sensitivity and specificity (Table 4).

For the Dataset 1 model (standardized autoantibody ratios), optimal thresholds for overall, benign, malignant, and stage I–II CMT classifications were 0.78, 0.60, 0.63, and 0.53, respectively (Table 4, top section). Assuming a CMT prevalence of 0.5 among hospital-referred, CMT-susceptible dogs (Zheng et al. 2022), the PPVs ranged from 0.741 to 0.751 and the NPVs from 0.751 to 0.906, indicating consistent predictive performance across disease stages.

The Dataset 2 model (MFI values) achieved particularly high NPV for overall CMTs (0.846) and high PPV for benign CMTs (0.806), supporting its potential use in clinical screening (Table 4, middle section). In contrast, the Dataset 3 model (combined standardized ratios and MFI values) showed moderate PPV and NPV values compared with individual models (Table 4, bottom section), suggesting that integrating raw and normalized inputs did not further enhance diagnostic reliability.

### Feature influence assessment in RF models

In RF classifiers, feature influence represents the cumulative reduction in Gini impurity contributed by a variable across all decision trees, rather than its direct correlation or direction of effect. A higher influence score indicates that the feature was more frequently used to partition mixed samples, thereby contributing more substantially to model purity.

**Figure 5.**
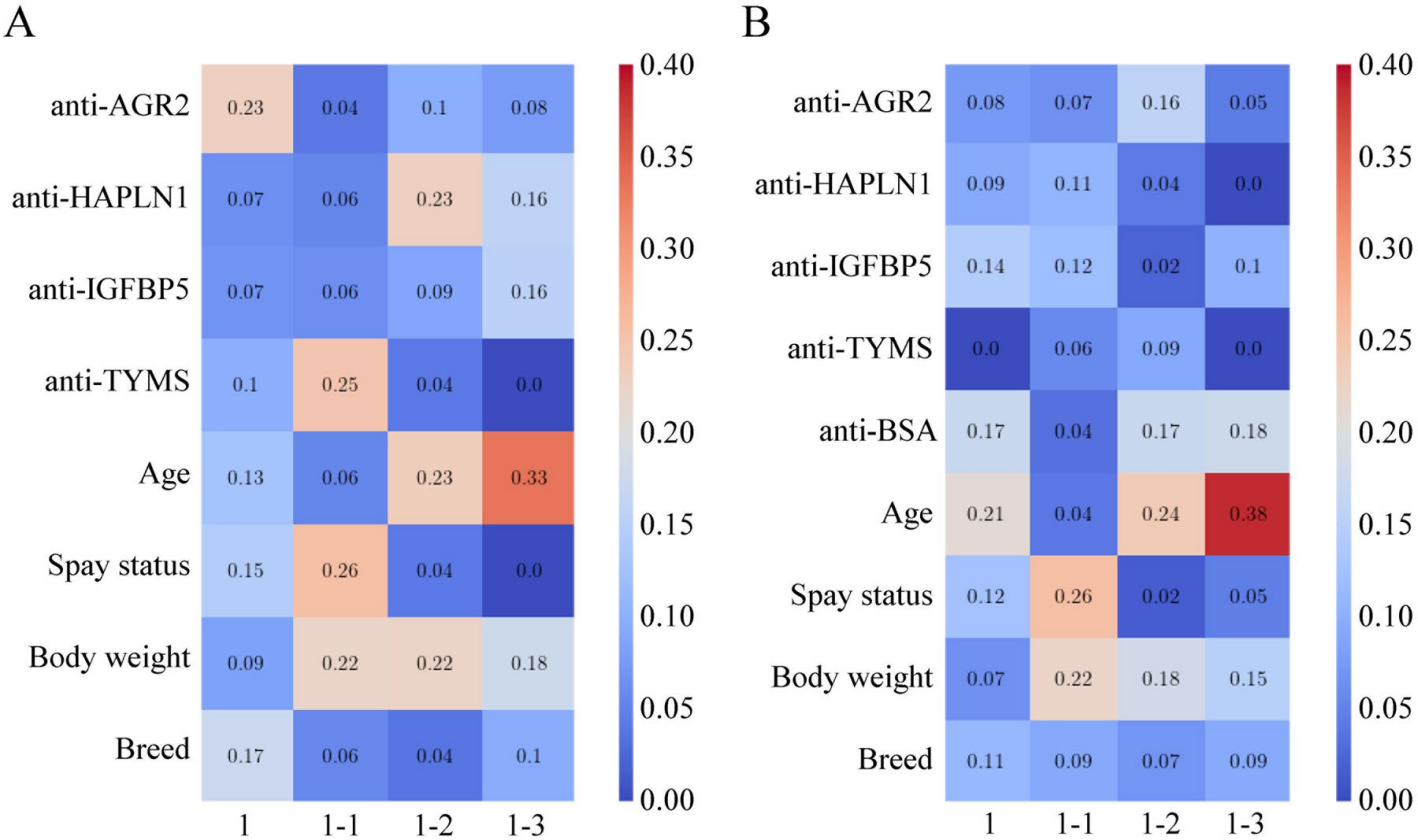
Feature influence across classification models. Heatmaps illustrate the relative influence of input features in RF models trained on (A) Dataset 1 (standardized autoantibody ratios) and (B) Dataset 2 (MFI values) for the following classifications: (1) healthy controls *vs*. all CMT cases, (1-1) healthy controls *vs*. benign CMTs, (1–2) healthy controls *vs*. malignant CMTs, and (1–3) healthy controls *vs*. stage I–II CMTs. Each cell represents the relative contribution of a feature to model performance, with warmer colors (red tones) indicating higher influence and cooler colors (blue tones) indicating lower influence.

Analysis revealed that the standardized ratios of anti-AGR2, anti-TYMS, and anti-HAPLN1 were the most influential features for classifying overall CMTs (score: 0.23), benign CMTs (0.25), and malignant CMTs (0.23), respectively (Figure 5A). None of the raw MFI values showed comparable influence, with scores ranging from 0.00 to 0.14 (Figure 5B). Among clinical variables, age contributed most to classifying early-stage malignancy (score: 0.33 in Figure 5A; 0.38 in Figure 5B), while spay status was a major determinant for benign CMT classification (0.26 in both Figure 5A and 5B).

## Discussion

Machine learning has been increasingly integrated into efforts to advance veterinary oncology, as highlighted by a recent scoping review (Li et al. 2025), with a strong emphasis on diagnostic applications, particularly for prevalent tumor types such as CMT. To date, the most mature applications involve image-based diagnostics, in which deep learning models have demonstrated high performance in tasks including tumor grading and malignancy characterization (Fragoso-Garcia et al. 2023; Xavier et al. 2024). In contrast, the use of serum-based biomarkers coupled with machine learning for CMT diagnostics remains relatively limited. One related study employed a machine learning algorithm incorporating age and serum thymidine kinase 1 (TK1) and C-reactive protein (CRP) levels to detect multiple canine cancers, including malignant mammary tumors (*n* = 28) (Sharif et al. 2025). Building upon this emerging but still underdeveloped area, the present study develops machine learning models based on a panel of serum autoantibodies integrated with common clinical variables, with the specific aim of discriminating early-stage CMTs from healthy controls and benign groups.

We previously identified TYMS, IGFBP5, HAPLN1, and AGR2 as CMT-associated antigens. TYMS catalyzes the synthesis of deoxythymidine monophosphate, driving DNA synthesis and cellular proliferation; its overexpression has been linked to poor prognosis and therapeutic resistance in multiple cancers (Song et al. 2021). IGFBP5 regulates mammary gland development and cancer cell migration, with context-dependent tumor-promoting activity (Waters et al. 2022). HAPLN1, a matrix-linking protein, contributes to extracellular matrix remodeling and tumor invasion (Wiedmann et al. 2023). AGR2, an endoplasmic reticulum (ER)-resident protein disulfide isomerase, supports protein folding, secretion, and tumor-associated stress adaptation (Yuan et al. 2024). Serum autoantibodies targeting these antigens have been associated with CMT development (Chang et al. 2021; Wu et al. 2025), supporting their potential as immunological biomarkers.

In the present study, we profiled a five-plex serum antibody panel—anti-AGR2, anti-HAPLN1, anti-IGFBP5, anti-TYMS, and anti-BSA—and evaluated its diagnostic utility in combination with clinical variables using machine learning models. Interestingly, raw MFI values for all tested autoantibodies were higher in clinically healthy controls than in CMT-bearing dogs. This apparently paradoxical finding aligns with reports of tumor-associated humoral immune suppression, characterized by impaired B-cell activation and diminished antibody production (Izhak et al. 2010; Stewart and Smyth 2011; Zhao and Fan 2025). Conversely, elevated baseline autoantibody titers in healthy individuals have been interpreted as a reflection of active humoral immune surveillance (Mair et al. 2022). Hence, the higher raw antibody levels in healthy dogs likely reflect stronger baseline immune responsiveness, including nonspecific antibodies such as anti-BSA IgG.

After normalization to anti-BSA levels, standardized ratios of anti-AGR2, anti-HAPLN1, anti-IGFBP5, and anti-TYMS were significantly higher in malignant and early-stage CMTs, suggesting selective amplification of tumor-specific immune responses despite global immune suppression. These findings imply that diagnostic value resides not in absolute antibody titers but in the relative magnitude of antigen-specific responses above baseline immunity. Consequently, normalization against individual background reactivity enhances the interpretability and reliability of humoral biomarkers, supporting the use of standardized ratios rather than raw MFI values for noninvasive CMT detection.

Epidemiological data from Mainland China reported that CMTs accounted for nearly half (46.7%) of all canine tumors between 2017 and 2021, with comparable distribution of benign (48.4%) and malignant (51.6%) lesions (Zheng et al. 2022). This high prevalence underscores the demand for reliable, noninvasive tools enabling early detection and timely intervention. In our study, diagnostic performance was further assessed using PPV and NPV under a presumed CMT prevalence of 0.5 among hospital-referred dogs. The RF model trained on standardized ratios achieved PPVs of 0.741–0.751 and NPVs of 0.751–0.906. The high NPV (0.906) indicates the potential utility of this model for excluding malignant CMTs in general screening, while the PPV (0.751) supports its application for triaging surgical candidates in conjunction with physical examination and fine-needle aspiration (FNA) before advanced imaging or histopathology. Collectively, the model based on standardized autoantibody ratios demonstrated balanced diagnostic performance, supporting its potential use as a noninvasive adjunct for CMT screening and pre-surgical triage in clinical practice.

Among the modeled predictors, the standardized ratios of anti-AGR2, anti-TYMS, and anti-HAPLN1 emerged as the most influential features for classifying overall, benign, and malignant CMTs, respectively. While RF modeling captures nonlinear and interactive effects, these feature influence scores represent statistical contributions rather than causal relationships and may be influenced by variable intercorrelations within the model. Biologically, the prominence of anti-AGR2 suggests that immune recognition of AGR2-expressing tumor cells reflects ER stress-related neoantigen exposure, whereas anti-TYMS responses may correspond to proliferative or DNA repair-linked antigenicity characteristic of early neoplastic transformation. The stronger influence of anti-HAPLN1 in malignant CMTs implies enhanced immune recognition of extracellular matrix remodeling and tissue invasion. In contrast, anti-IGFBP5 contributed minimally across all models, suggesting limited diagnostic discrimination within this specific biomarker set despite the biological significance of IGFBP5 expression in mammary epithelium.

Among clinical variables, age and spay status—both established epidemiological determinants of CMT risk (Vascellari et al. 2016; Gray et al. 2020)—also contributed to classification performance. Age and spay status were most relevant to malignant (overall and stage I–II) and benign CMTs, respectively, but were not consistently retained in models trained on standardized ratios. Because age distributions were comparable among healthy controls (median = 8 years; range = 2–17), benign CMTs (8.4; 2–16), and malignant CMTs (10; 1–16), and serum autoantibody levels showed no significant association with either variable (Chang et al. 2021), these results suggest that autoantibody-based biomarkers reflect tumor-specific immune responses rather than demographic confounders. This reinforces their potential to complement established risk factors and enhance diagnostic precision in CMT detection.

Several limitations should be acknowledged. First, recruitment from a single institution and the relatively small number of healthy controls may constrain generalizability and introduce class imbalance. The limited availability of healthy controls reflects a common challenge in veterinary datasets, as healthy animals rarely undergo diagnostic testing. To mitigate this issue, all models were evaluated using five-fold cross-validation, ensuring each subset served once as a test set. This approach minimizes bias arising from specific data distributions and provides a reliable estimate of model stability. Consistent accuracy across folds indicated no overfitting and demonstrated acceptable model stability. While class imbalance may reduce sensitivity for healthy cases, cross-validation provides a reasonable safeguard for robustness and reproducibility. Nevertheless, external validation using independent, multi-institutional cohorts will be essential to confirm reproducibility and clinical applicability.

Second, while this study focused on antibodies targeting four well-characterized oncogenic antigens, the broader autoantibody repertoire in CMTs remains to be defined. Future studies employing proteomic or high-throughput antibody discovery platforms could identify additional biomarkers to improve diagnostic precision and delineate tumor biology. Integrating serological, genetic, and imaging-derived features may further refine predictive modeling and enhance translational potential.

In conclusion, this study establishes proof-of-concept for a machine learning-assisted multiplex autoantibody assay as a noninvasive approach for detecting and stratifying CMTs. Although the model demonstrated moderate diagnostic accuracy, its high NPV underscores its potential for screening and early exclusion of malignancy. Continued refinement, larger-scale validation, and integration with cytological and imaging assessments will be critical for translating this approach into routine veterinary oncology practice.

## Supplementary Material

Supplementary Tables.docx
