# Supplementary material for: Machine learning-assisted detection of canine mammary tumors using serum autoantibody signatures
Source: Vet Q. 2026 Jan 21;46(1):2617470. doi: 10.1080/01652176.2026.2617470 (PMC12825649; doi:10.1080/01652176.2026.2617470)
Supplement: Supplementary Tables.docx [file TVEQ_A_2617470_SM7111.docx]

| Table S1. Diagnostic performance of serum autoantibody levels in distinguishing canine mammary tumors from healthy controls | | | | |
| --- | --- | --- | --- | --- |
| Serum autoantibodies | The area under ROC curve, AUC (95% confidence interval) | | | |
|  | CMTs  (*n* = 154) | Benign CMTs  (*n* = 31) | Malignant CMTs  (*n* = 123) | Stage I-II  (*n* = 73) |
| MFI values^a^ | | | | |
| Anti-AGR2 | 0.618 (0.521 ‒ 0.724) | 0.661 (0.520 ‒ 0.789) | 0.607 (0.501 ‒ 0.709) | 0.593 (0.471 ‒ 0.703) |
| Anti-HAPLN1 | 0.583 (0.475 ‒ 0.695) | 0.573 (0.438 ‒ 0.701) | 0.585 (0.473 ‒ 0.697) | 0.583 (0.460 ‒ 0.698) |
| Anti-IGFBP5 | 0.638 (0.537 ‒ 0.729) | 0.674 (0.539 ‒ 0.803) | 0.629 (0.533 ‒ 0.734) | 0.638 (0.529 ‒ 0.745) |
| Anti-TYMS | 0.614 (0.521 ‒ 0.705) | 0.596 (0.455 ‒ 0.724) | 0.618 (0.520 ‒ 0.709) | 0.605 (0.492 ‒ 0.716) |
| Anti-BSA | 0.665 (0.574 ‒ 0.754) | 0.639 (0.509 ‒ 0.766) | 0.671 (0.574 ‒ 0.769) | 0.662 (0.546 ‒ 0.762) |
| Standardized ratios (normalized with anti-BSA)^b^ | | | | |
| Anti-AGR2 | 0.665 (0.574 ‒ 0.759) | 0.608 (0.459 ‒ 0.743) | 0.679 (0.573 ‒ 0.773) | 0.678 (0.569 ‒ 0.776) |
| Anti-HAPLN1 | 0.698 (0.601 ‒ 0.793) | 0.659 (0.532 ‒ 0.782) | 0.707 (0.610 ‒ 0.799) | 0.699 (0.591 ‒ 0.798) |
| Anti-IGFBP5 | 0.642 (0.537 ‒ 0.740) | 0.592 (0.447 ‒ 0.720) | 0.654 (0.560 ‒ 0.755) | 0.648 (0.542 ‒ 0.753) |
| Anti-TYMS | 0.685 (0.588 ‒ 0.778) | 0.637 (0.501 ‒ 0.762) | 0.697 (0.595 ‒ 0.790) | 0.692 (0.586 ‒ 0.795) |
| ^a^ Autoantibody levels are represented as median fluorescence intensity (MFI) values.  ^b^ Standardized ratios were calculated by dividing the MFI of each autoantibody by the MFI of anti-BSA within the same sample. | | | | |

| Table S2. Sensitivities and specificity of serum autoantibodies for detecting CMTs *vs*. healthy controls | | | | |
| --- | --- | --- | --- | --- |
| Serum autoantibodies | Performance metrics (sensitivity / specificity) for predicting positive cases^a^ | | | |
|  | CMTs  (*n* = 154) | Benign CMTs  (*n* = 31) | Malignant CMTs  (*n* = 123) | Stage I‒II  (*n* = 73) |
| MFI values^b^ | | | | |
| Anti-AGR2 | 0.462 / 0.786 | 0.769 / 0.516 | 0.462 / 0.780 | 0.462 / 0.795 |
| Anti-HAPLN1 | 0.359 / 0.864 | 0.359 / 0.839 | 0.359 / 0.870 | 0.359 / 0.877 |
| Anti-IGFBP5 | 0.718 / 0.532 | 0.795 / 0.516 | 0.718 / 0.520 | 0.718 / 0.548 |
| Anti-TYMS | 0.795 / 0.455 | 0.795 / 0.419 | 0.795 / 0.463 | 0.795 / 0.425 |
| Anti-BSA | 0.564 / 0.701 | 0.795 / 0.452 | 0.564 / 0.724 | 0.564 / 0.712 |
| Standardized ratios (normalized with anti-BSA)^c^ | | | | |
| Anti-AGR2 | 0.747 / 0.564 | 0.677 / 0.564 | 0.764 / 0.564 | 0.767 / 0.538 |
| Anti-HAPLN1 | 0.695 / 0.641 | 0.903 / 0.436 | 0.715 / 0.641 | 0.890 / 0.462 |
| Anti-IGFBP5 | 0.701 / 0.590 | 0.839 / 0.359 | 0.732 / 0.590 | 0.740 / 0.562 |
| Anti-TYMS | 0.721 / 0.615 | 0.677 / 0.615 | 0.732 / 0.615 | 0.753 / 0.615 |
| ^a^ Sensitivity and specificity were determined using the optimal cutoff based on the Youden Index (Youden's J statistic).  ^b^ Autoantibody levels are represented as median fluorescence intensity (MFI) values.  ^c^ Standardized ratios were calculated by dividing the MFI of each autoantibody by the MFI of anti-BSA in the same sample. | | | | |
